# Checkpoint antibody receptor modified ARMed CAR T circumvents the suppressive immunome in GBM

**DOI:** 10.3389/fimmu.2025.1579925

**Published:** 2025-07-31

**Authors:** Danielle R. Cook, Alina C. Boesteanu, Yibo Yin, Reiss Reid, Laura Roccograndi, Nadia Dahmane, Maria Martinez-Lage, Donald M. O’Rourke, Carl H. June, Laura A. Johnson

**Affiliations:** ^1^ Center for Cellular Immunotherapies, Perelman School of Medicine, University of Pennsylvania, Philadelphia, PA, United States; ^2^ Glioblastoma Translational Center of Excellence, Abramson Cancer Center and Department of Neurosurgery, Perelman School of Medicine, University of Pennsylvania, Philadelphia, PA, United States; ^3^ Department of Neurosurgery, Perelman School of Medicine, University of Pennsylvania, Philadelphia, PA, United States; ^4^ Department of Pathology and Laboratory Medicine, Perelman School of Medicine at the University of Pennsylvania, Philadelphia, PA, United States

**Keywords:** chimeric antigen receptor (CAR), tumor microenvironment (TME), glioblastoma (GBM), checkpoint inhibition (CPI), fourth generation CAR, ARMed CAR, minibody, antibody-secreting CAR

## Abstract

**Introduction:**

Glioblastoma (GBM) remains a deadly cancer with non-curative upfront treatment of radiation, resection, and chemotherapy. Not only has the standard of care for GBM patients not improved significantly over the past decade, life expectancy is less than 18 months, with no standard second-line therapy. We previously developed a 2^nd^ generation 4-1BB co-stimulated chimeric antigen receptor (CAR) targeting tumor-specific variant of the epidermal growth factor receptor (EGFRvIII) for treating patients with GBM. This CAR T was used in Phase 1 clinical trials, and demonstrated that CAR T cells rapidly trafficked to tumors and showed initial anti-tumor activity upon encountering EGFRvIII-bearing tumor cells. However, the CAR T cells rapidly became exhausted, losing anti-tumor function, with no durable objective tumor responses.

**Methods:**

Here, we evaluated the GBM immune environment in a syngeneic implantable GL261 murine model. Prior to tumor implantation, brain-resident immune cells were mostly absent. Following tumor engraftment, there was a pronounced increase in immune cell infiltration over time and with GBM size. Immune infiltrates were intitally comprised of early-arriving lymphocytes including T, NK, and B cells, later this shifted towards increased presence of macrophages and myeloid-derived suppressor cells. Evaluating both fresh and archival GBM samples from patients, we found similarly high levels of infiltrating immune cells, and PDL1 expression on both tumor and immune cells. PD1/PDL1-antibody (Ab) mediated checkpoint inhibition (CPI) has been transformative in treating several types of solid tumors; however the localization of GBM behind the blood-brain barrier limits Ab access, and CPI trials have been unsuccessful in treating GBM. To deliver PD1/PDL1 checkpoint Ab for patients with GBM, we engineered our EGFRvIII-targeted CAR T cells to function as bio-factories, producing and secreting anti-PD1 mini-Abs *in situ* at the site of GBM.

**Results:**

These Ab receptor-modified (ARMed) CAR T cells produced functional PD1 minibodies in vitro and demonstrated anti-tumor activity *in vivo* in a GBM xenograft model using NOD-Scid gammaC-null (NSG) mice. Delivered systemically, both soluble Ab plus CAR T, and ARMed CAR T cells improved subcutaneously implanted GBM treatment over CAR T alone, while treatment of orthotopic GBM treatment was only improved with ARMed CAR T therapy.

**Discussion:**

These findings demonstrate that engineering EGFRvIII-directed CAR T cells to secrete checkpoint inhibitors locally can overcome immunosuppressive barriers in GBM and bypass the limitations of systemic antibody delivery. This strategy enhances CAR T cell functional persistence and holds strong translational potential for treating GBM and other CNS-localized disease.

## Introduction

Glioblastoma (GBM) is the most aggressive and common type of primary malignant brain tumor in adults. It carries a poor prognosis, with a median survival of 15 to 18 months. Despite aggressive treatment, including resection, radiation, and chemotherapy, patients often experience rapid tumor recurrence and disease progression (1). Challenges in GBM treatment include the infiltrative nature of the tumor, tumor cell heterogeneity, development of resistance to therapy, a complex tumor microenvironment (TME), and low levels of tumor mutations, resulting in few tumor-specific T cells (2). Immune cell infiltration in the GBM microenvironment has been implicated in tumor progression via suppression of tumor-specific T cells (3–7). With recent clinical advances in adoptive transfer T cell therapy, including chimeric antigen receptor (CAR) T cells, the role of TME-induced immune suppression in GBM has become a focus, highlighting intratumoral complexity and its impact on immunotherapy treatment (8–10).

Recent clinical trials including our own, have attempted to provide a T cell response to GBM by infusing patients with autologous CAR T cells targeted to GBM-specific antigens (6, 11–13). These studies demonstrated rapid early anti-tumor activity, including an influx of activated T cells into the tumor and radiologic evidence of tumor regression within 24 hours of infusion (6, 13). However, these responses were mostly transient as tumors recurred and progressed rapidly, coinciding with a loss of T cell function and increased markers of immunosuppression. These findings suggest that while CAR T cell therapy facilitates trafficking of tumor-specific T cells to the tumor site, including across the blood-brain barrier (BBB), and that these T cells recognize and eliminate antigen-bearing cells, this anti-tumor function is generally short-lived.

Programmed cell death-1 (PD1) on T cells, and programmed death-ligand 1 (PDL1) on tumors comprise an immunosuppressive axis within the TME and have been shown to play a pivotal role in GBM progression (14, 15). PD1-PDL1 interaction inhibits T cell proliferation, cytokine production, and cytotoxic activity, allowing tumors to escape T cell-mediated destruction. PDL1 expression has also been identified on tumor-infiltrating myeloid cells that secrete the immunosuppressive cytokine IL-10 in GBM (16). Clinically, GBM has been shown to upregulate PDL1 in response to CAR T cells, potentially driving an exhausted T cell phenotype and diminished antitumor immune response (6, 17, 18). A meta-analysis of PDL1 expression in GBM found that PDL1 expression was associated with worse overall survival, supporting a potential immunosuppressive mechanism at work (19). Consequently, targeting the PD1-PDL1 pathway has emerged as a promising therapeutic strategy to enhance immune-mediated tumor clearance in GBM patients.

Clinically, the administration of antibodies (Abs) inhibiting PD1-PDL1 signaling (CPI) has been effective in treating some solid tumor indications, including melanoma, lung, and bladder cancer (20–22). However, CPI trials in GBM have failed to benefit patients, potentially due to the lack of endogenous tumor-reactive T cells and limited Ab ability to cross the BBB (5, 14, 23–25). Addressing the lack of endogenous tumor-specific T cells, our team recently investigated EGFRvIII CAR T in combination therapy with the anti-PD1 Ab pembrolizumab, in patients with GBM. Similar to treatment with CAR T alone, this trial showed no clinical anti-tumor response (6, 26). From these CAR T trials we have learned that engineered CAR T cells can cross the BBB and home to the tumor site in the brain, and that PDL1 is upregulated after CAR T treatment, suggesting a possible role in the loss of CAR T cell function in GBM and that providing CPI Ab is not sufficient to improve the therapy. To overcome this limitation, we have engineered fourth generation CPI Ab receptor-modified (ARMed) EGFRvIII CAR T cells, capable of trafficking into GBM to produce and secrete anti-PD1 Abs *in situ*, and evaluated them *in vitro* and *in vivo* in subcutaneous and orthotopic GBM murine models.

## Results

### GBM demonstrates a macrophage-enriched immune cell infiltration in the tumor microenvironment in an experimental murine model

**Figure 1:**
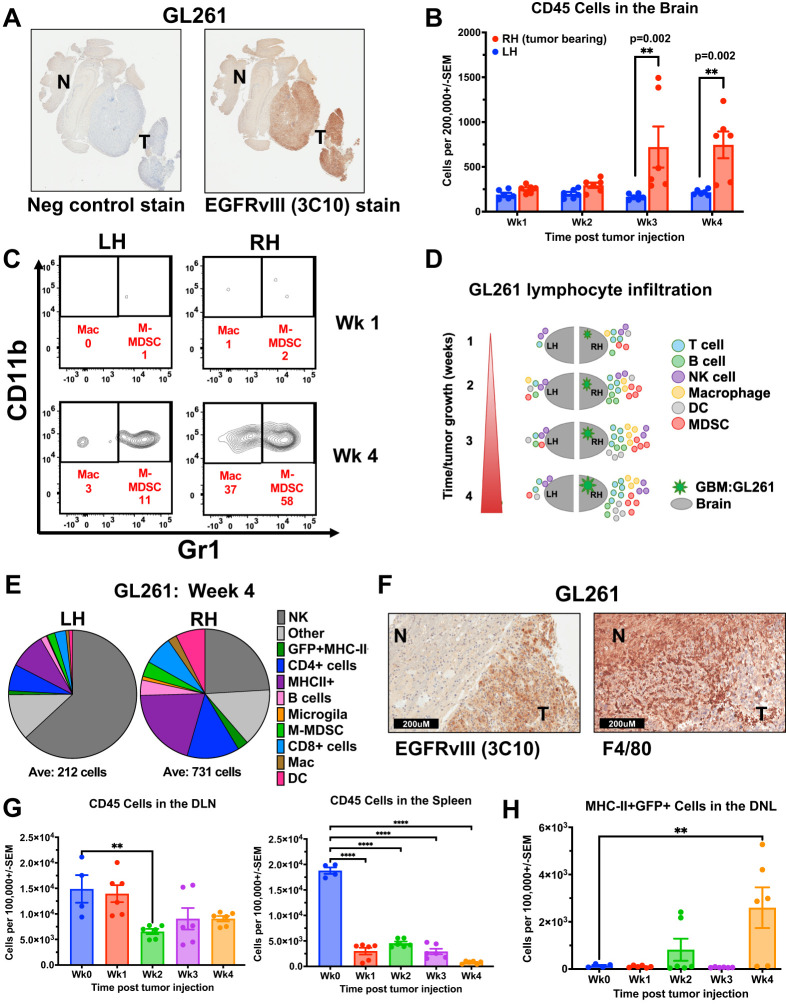
Immune cell infiltration and kinetics in murine GBM model increase with tumor engraftment. **(A, F)** Immunohistochemistry (IHC) staining of FFPE orthotopic murine GL261 GBM tumor sections from C57Bl/6 mouse **(A)** EGFRvIII staining using anti-EGFRvIII Ab (clone 3C10) and negative control secondary Ab alone. “T” designates tumor and “N” designates normal tissue. (40X magnification) **(B-E, G)** Fresh tumors were collected over 4 weeks from mice previously injected intracranially (IC) with syngeneic GL261 GBM cell line and processed as single-cell suspensions before Ab staining of cellular differentiation markers and flow cytometry analysis. **(B)** Quantification of CD45-positive immune cells in the ipsilateral right hemisphere (RH, tumor-bearing) versus contralateral left hemisphere (LH) brains of GL261-tumor bearing mice (n=3 mice per time point, stained in technical replicates) weekly for 4 weeks following IC tumor injection. Mean ± std err is shown. **(C)** Representative flow cytometry density plots of CD45 and F4/80-positive cells by CD11b and Gr1 expression, with CD11b+Gr1- representing macrophages and CD11b+Gr1+ as M-MDSCs in RH versus LH brains of mice at one and four weeks after GL261 tumor injection. **(D)** Schematic representation of immune cell infiltration dynamics over time in the GL261 orthotopic murine GBM model, separated by hemisphere. **(E)** Pie chart depiction of the relative proportions (averages) of immune cell types in the tumor-bearing RH and LH four weeks post-tumor injection. Below the pie chart is the average of CD45+ cells per 200,000 total cells. **(F)** IHC staining of FFPE orthotopic GL261 tumors stained with EGFRvIII Ab (clone 3C10), or F4/80 Ab. “T” designates tumor and “N” designates normal tissue. **(G)** Bar graph of CD45 cells in the draining lymph nodes (DLN, n=3 mice per time point, stained in technical replicates), and spleens (n=3 mice per time point, stained in technical replicates) of tumor-bearing mice compared with non-tumor-bearing wild-type control mice (Wk 0; n = 4). Mean ± std err are shown. **(H)** Bar graph of MHC-II+GFP+ cells in the DLN (n=3 mice per time point, stained in technical replicates) of tumor-bearing mice compared with non-tumor-bearing wild-type control mice (Wk 0); n = 4). Mean ± std err are shown. Cell marker phenotype flow cytometry gating used to generate D and E is supplied in **Supplementary Figure 1**. Statistical analyses were conducted by Multiple Mann-Whitney in **(B)**, and One-way ANOVA in **(G, H)**; Statistical significance was denoted as follows: P < 0.01 (**) and P < 0.0001 (****).

To understand the composition of the immune components within the GBM TME, we used a syngeneic implantable orthotopic murine GBM (EGFRvIII+ GFP+ GL261) in immunocompetent C57Bl/6 mice (27) (**Figure 1A**). Tumor cells were implanted in the right brain hemisphere in 4 groups of mice, to engraft and expand for 4 weeks. Each week after implantation, one group of mice was sacrificed, and brains were divided into hemispheres. Each hemisphere was processed as a single-cell suspension, enumerated, and stained with Abs for cellular differentiation markers, and analysed by flow cytometry (**Supplementary Figures S1A, B**). Results identified immune infiltrates by mCD45 expression (**Figure 1B**), then further characterized into different immune cell populations (**Figure 1C**, **Supplementary Figure S1C**). We found that as early as the first week after tumor implantation, there were infiltrating CD45+ immune cells, mainly in the tumor-side (ipsilateral) right hemisphere (RH), compared with the contralateral left hemisphere (LH), and these infiltrates continued to increase over time following GBM engraftment (**Figure 1D**, **Supplementary Figure S1C**). By the end of week 4, at the conclusion of the experiment, the non-tumor bearing LH showed low levels of immune cells (**Supplementary Figure S1C**). In contrast, the tumor-bearing RH exhibited significant infiltration of different immune cells, increasing in quantities compared to week one. These infiltrates included CD4+ T cells, MHC-II+ cells (which included some myeloid-derived suppressor cells (MDSCs), although they are not shown separately), monocyte-derived MDSCs (M-MDSCs), CD8+ T cells, and dendritic cells (DCs) (**Figure 1E**, **Supplementary Figure S1C**), however, microglia (F4/80+CD45lo) levels remained relatively constant throughout. The high proportion of macrophages and M-MDSC in tumor immune infiltrates was confirmed *in situ* by F4/80 immunohistochemistry (IHC) staining of Formalin-Fixed Paraffin-Embeded (FFPE) tumor tissue (**Figure 1F**). Importantly, microglia are also F4/80+, leading to an overall low-level positive (brown) staining across the brain and tumor sections. All immune infiltrates increased their total cell numbers over each of the four weekly timepoints evaluated. Notably, the spleen but not draining lymph nodes (DLN) from each mouse at the same time-points showed a decrease in CD45+ cells from pre-tumor baselines, as they increased in the brain, supporting a potential re-localization of immune cell populations (**Figure 1G**). Importantly, there was an increase in MHC-II+GFP+ cells in the draining lymph nodes (DLN) by week four, suggesting that antigen-presenting cells had taken up tumor (GL261 GFP+) in the brain and trafficked to the DLN (**Figure 1H**).

### Clinically GBM contains a rich microenvironment comprised of various infiltrating immune cell types

**Figure 2:**
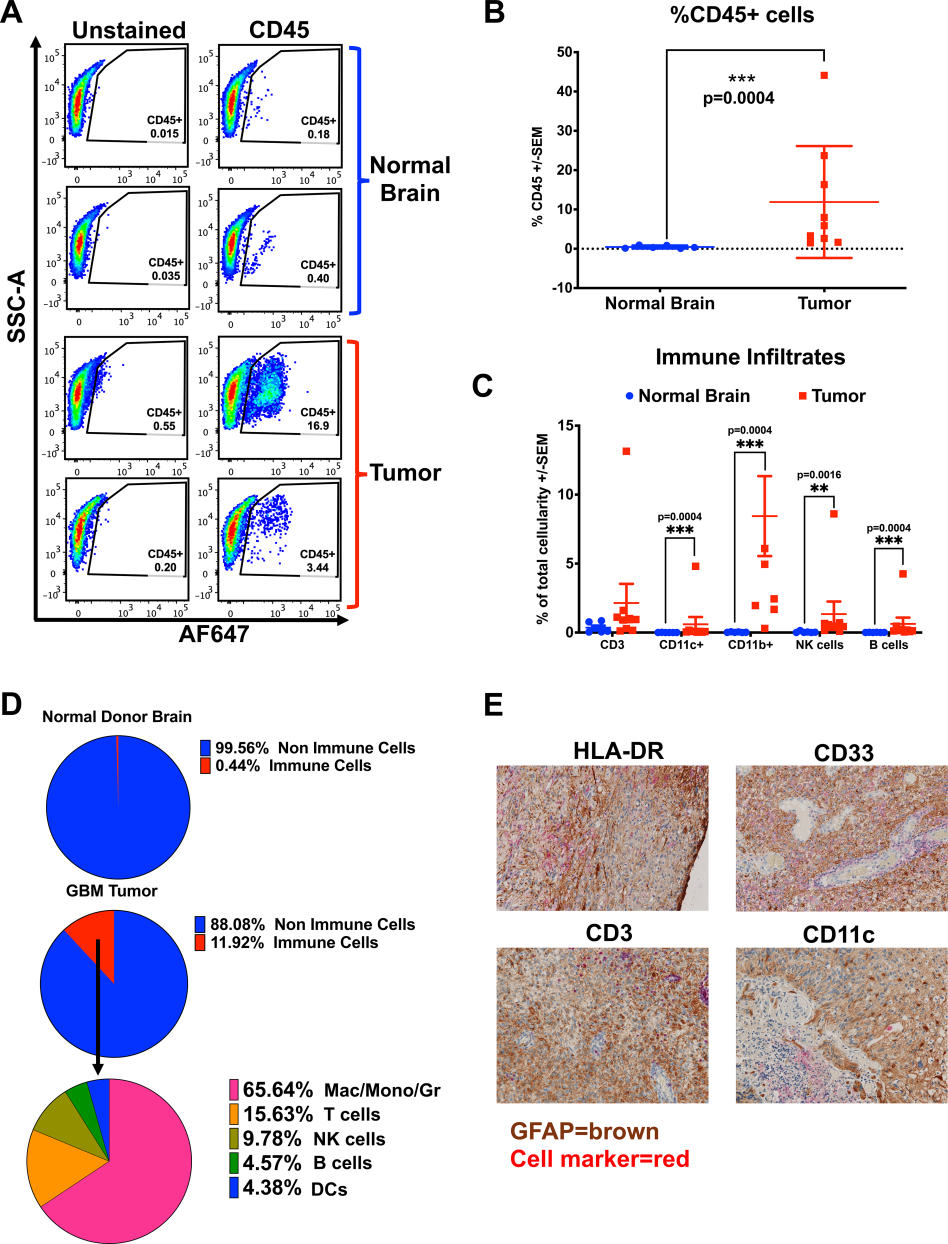
Patient GBMs contain diverse populations of immune infiltrates. **(A-D)** Fresh GBM samples were prepared as single-cell suspensions, washed, enriched for CD45-positive cells by positive selection with magnetic beads (Miltenyi), and stained with cellular differentiation Abs to identify different immune cell populations. **(A)** Representative flow cytometry dot plots of percent live CD45-positive cells in brains of normal donors versus patient GBM samples. **(B)** A graph of the percentage of live CD45-positive cells, stained and analyzed by flow cytometry from the brains of normal donors (n = 6) and GBM patient samples (n = 9), Mean ± std err. **(C)** Graph of percentage total cellularity comprising of each immune cell type, from the brains of normal donors (n = 6) *vs* patient GBM samples (n = 9), Mean ± std err. Note, for CD11b+, three patient values are not shown, offscale at greater than 15%. **(D)** Pie charts depicting the average immune cell percentages across samples (live CD45-positive cells), and non-immune cells in patients’ GBMs (n = 9) and the brains of normal donors (ND), (n = 6). Within the GBM patient samples, the contribution of each immune cell type is shown (averages of 9 samples). **(E)** Immunohistochemical staining of archival human GBM FFPE tumor sections with GFAP (tumor marker) in brown chromogen, co-stained to demonstrate infiltrating immune cells in red chromogen, with Abs against HLA-DR (MHC-II), CD33 (myeloid cells), CD11c (pan-dendritic cells), or CD3 (T cells) with hematoxylin counter-staining (100X magnification). Cell marker phenotype flow cytometry gating used to generate **(C, D)** is supplied in **Supplementary Figure 2**. Statistical analyses were conducted using the multiple Mann-Whitney test, unpaired, non-parametric; statistical significance was denoted as follows: P < 0.01 (**) and P < 0.001 (***).

To compare the immune infiltrates in the murine GBM model with those in patients, we used a 13-color flow cytometry panel of Abs to evaluate immune cells from 9 freshly resected patient GBM samples and 6 control samples of non-GBM donor brains obtained from neurosurgical resections at the Hospital of the University of Pennsylvania (**Supplementary Figures S2A, B**). Fresh tumors or normal brain samples were processed into single-cell suspensions prior to magnetic bead enrichment for CD45-positive cells, allowing us to enrich for CD45-hi immune infiltrates (28). The CD45-positive cells were cryopreserved prior to further analysis, and the CD45-negative fraction (enriched for tumor) was plated to establish patient-derived GBM cell lines, then banked. A small portion of each sample was stained with CD45 Ab and analyzed to determine the percentage of immune cells in each sample prior to enrichment, allowing a quantitative flow cytometry analysis based on total tissue cellularity. Tumors contained varied proportions of immune cells, while normal brain tissue controls uniformly had very few (**Figures 2A, B**). As shown in **Figure 2B**, patient GBM samples had significantly more immune cells than the normal brain (mean 11.89% versus 0.44%, respectively, p = 0.0004). The CD45-enriched immune infiltrates were stained according to our panel of cellular-differentiation Abs and analyzed by flow cytometry (**Supplementary Figures S2A, B**). Based on cellular staining profiles, we assigned each to immune cell subtypes (DCs as CD11c+, Mac/Mono/Gr as CD11b+, T cells as CD3+, NK cells as CD56+, and B cells as CD19+) and found all were enriched in GBM compared with normal brain tissue (**Figure 2C, D**). This data supports that the BBB does not prevent immune cell infiltration in human GBM. **Figure 2D** shows the relative proportions of immune cell types from the average of normal brain and from GBM, with Mac/Mono/Gr cells comprising the majority of GBM infiltrates (65.64%), followed by smaller populations of T cells (15.63%) that were almost exclusively CD4+, and NK cells (9.78%), with the smallest populations comprised of B cells (4.57%) and DCs (4.38%). We confirmed the presence of these immune infiltrates *in situ* by IHC staining on archival FFPE GBM samples, demonstrating extensive MHC-II (HLA-DR+), present on DCs and macrophages, T cells (CD3+), macrophage/myeloid cells (CD33+) and DCs (CD11c+) distributed throughout (**Figure 2E**). Together, these show similar results to the composition of immune infiltrates found in the syngeneic orthotopic GBM mouse model, with high levels of MHC-II+ cells, DCs and T cells that are absent in non-tumor-bearing brain, suggesting that the presence of GBM recruits immune cells.

To determine whether the immune cells in the TME resulted from specific recruitment or were simply a reflection of those cells present in circulation, we collected 8 PBMC samples patient-matched to the fresh GBM resections, and 7 normal donor PBMC (**Supplementary Figure S3**). Between GBM patients and normal donor PBMC, we found a significant reduction in the overall percentage of CD45+ cells circulating in patients compared with normal donors (**Supplementary Figure S3A**). Additionally, the composition of immune cells differed, with GBM patient PBMC showing a relative increase in CD11b+ and CD11c+ and a reduction of T cells (CD3+) compared with normal donors (**Supplementary Figure S3B**). In PBMC from GBM patients, the highest proportion of immune cells in the periphery on average were DCs (36%) compared with 4.4% in tumors, B cells were present at 15% in PBMC versus 4.3% in tumors, while NK cells comprised just 1% of PBMC with 9.7% in tumors (**Supplementary Figure S3B**); the vast majority (65.8%) of immune cells in tumor were Mac/Mono/Gr cells but these cells comprised only 32% of patient PBMC (**Figures 2D**, **Supplementary Figure S3B**). Next, we evaluated CD4 and CD8 T cells in all samples. In the GBM immune infiltrates, most T cells were CD4 helper T cells (1.2% of total cellularity), while CD8 T cells were sparse (0.011%). Reflecting a lower overall immune infiltration, normal donor brain tissue contained fewer T cells overall, with CD4 T cells at 0.3% and no detectable CD8 T cells (0.000%) (**Figures 2D**, **Supplementary Figure S3C**).

In the PBMC, GBM patients and normal donors showed similar ratios of CD4 to CD8 T cells (range 1.2-2.1); however, GBM patients’ PBMC contained substantially fewer peripheral T cells overall, with CD4 at 10% and 33.6%, and CD8 at 5.8% and 17.5%, in GBM versus ND respectively (**Supplementary Figure S3C**). In normal donors, T cells were the predominant PBMC population, mirroring their relative abundance among those few immune cells found in normal brain. In GBM patients, T cells comprised a similar proportion (about 15%) in both blood and tumor, while Mac/Mono/Gr (32%) and DCs (36%) equally predominated in the blood. The TME contained almost twice as many Mac/Mono/Gr (64%), with few DCs (4.6%) compared with blood (**Supplementary Figure S3B**), suggesting a selective enrichment of specific immune cell populations within the tumor.

In comparing the TME in GBM between an immune-intact syngeneic murine model of GBM and patients, the mouse model reproduced several aspects of the infiltration and composition of immune cells found in patients, however, there were a few notable differences. Of relevance, the time for tumors to develop into clinical disease is substantially reduced in mouse models, from days to weeks in mice versus years to decades in patients. Additionally, the types of infiltrates were substantially different between patients and mice in at least one aspect, in that T cells made up almost 50% of the infiltrates in the mouse model, with about one-quarter of those being CD8+, while in GBM patient samples, the total T cells typically comprised closer to 15%, with less than 1% CD8+ (**Figures 1E**, **2D**). This result is not entirely surprising, as the methylcholanthrene-induced murine GL261 GBM is known to express high levels of non-synonymous tumor mutations that give rise to endogenous tumor-reactive T cells, unlike the low tumor mutation burden in naturally arising GBM (29, 30).

### GBMs and their immune infiltrates express high levels of PDL1

**Figure 3:**
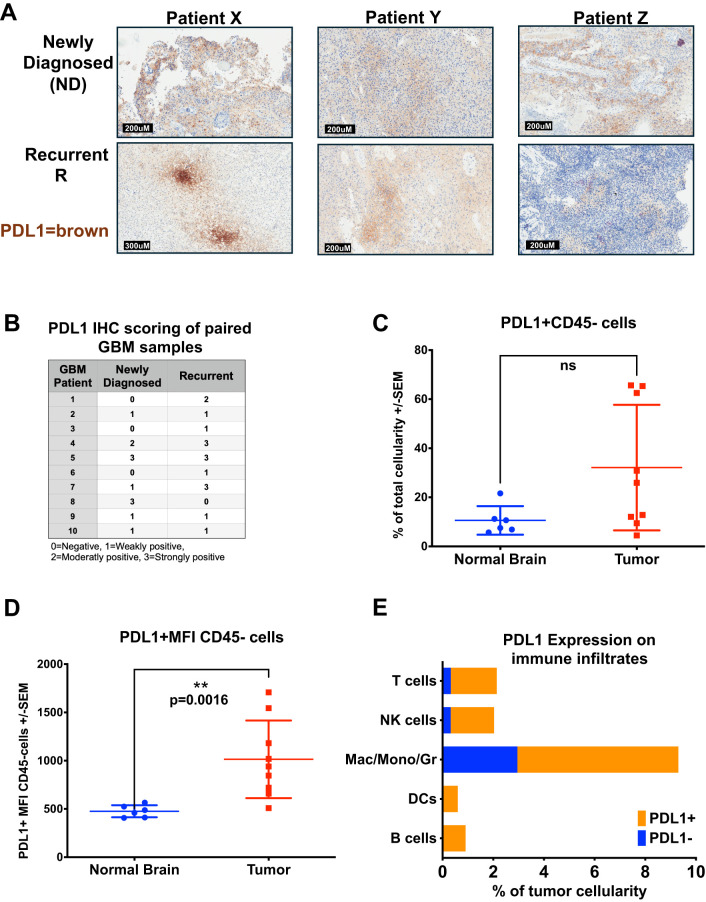
PDL1 is widely expressed in Human GBM and Immune Infiltrates. **(A, B)** GBM patients with matched newly diagnosed and recurrent tumors had FFPE sections stained for PDL1. **(A)** Representative IHC staining of FFPE GBM tumor sections from three patients (X,Y, and Z) with matched newly diagnosed and recurrent tumors were stained for PDL1 in brown chromogen (hematoxylin counter-stain). **(B)** Table of PDL1 IHC scoring from 10 patients with samples of both newly diagnosed and recurrent disease on a scale of 0-3, assessed by a neuropathologist. **(C–E)** Fresh GBM or normal brain cell suspensions from **Figure 2** were stained for PD1 and PDL1 in addition to cellular differentiation markers. **(C)** Percent cellularity of PDL1-positive cells within the live CD45-negative cell population in brains of normal donors (n = 6) versus those from GBM patients (n = 9), analyzed by flow cytometry (p = n.s.). **(D)** Mean PDL1 fluorescence intensity of PDL1-positive cells within the live CD45-negative cell population in brains of normal donors (n = 6) versus GBM patients (n = 9), analyzed by flow cytometry. **(E)** CD45-positive immune cells from GBMs were also stained with PD1 and PDL1, results shown in a stacked bar graph comparing the average percent cellularity of PDL1-positive cells within each immune subset (n = 9), analyzed by flow cytometry. Statistical analyses were conducted using the multiple Mann-Whitney test, unpaired, non-parametric; statistical significance was denoted as follows: P < 0.01 (**), and P ≥ 0.05 was considered not significant (ns).

Building on previous descriptions of PDL1 in GBM we evaluated PDL1 expression by IHC in 10 pairs of archival patient-matched newly diagnosed (ND) and recurrent FFPE GBM samples (**Figures 3A, B**) (18). We found heterogeneity of PDL1 expression levels between samples, with intensity of staining rated on a scale of 0-3, with 0 denoting negative for staining, and 3 as strongly positive. Different staining patterns were observed and were heterogeneous, homogeneous, or focally positive, and varied across the 20 tumors evaluated. The focal staining in **Figure 3A**, patient X recurrent tumor section, resembles a staining pattern associated with localized T cell activity with interferon-gamma (IFNγ) inducing *in situ* upregulation of PDL1 (31). In this evaluation, the majority of GBM samples stained at least weakly positive for PDL1, with 7/10 ND and 9/10 recurrent GBM with detectable staining. In 4 cases, there was equivalent expression in ND and recurrent GBM from the same patient; 5 increased PDL1 from ND to recurrent, while in only one case, PDL1 expression decreased from ND to recurrent disease. Generally, PDL1 expression was low in these samples, with only 20% ND and 30% recurrent GBM staining strongly positive. Previous research involving PD1-PDL1 interaction has mainly focused on PDL1 expression on tumor cells (32–35). However, immune cells can also be a source of PDL1, though the functional impact of this in the GBM TME remains unknown (36).

To evaluate whether immune infiltrating cells could contribute to PD1-PDL1-mediated immunosuppression and T cell dysfunction in GBM, we looked at PD1 and PDL1 expression on patient tumors and each immune cell subset from the fresh GBM samples. In both GBM and normal donor brain CD45-negative cell fractions, presumably comprised of tumor or normal brain cells, we saw low percentages of PDL1-positive cell staining from normal donors (mean 10.6%), with higher percentages of GBM (32%) staining PDL1 positive, though the GBM samples showed high variability, with between 5-70% (**Figure 3C**). However, the mean fluorescent intensity (MFI) between the two groups was significantly different, indicating the levels of PDL1 proteins on the non-CD45 cells was increased in GBM patient samples compared with normal donor brain, MFI of 476 in ND versus MFI of 1014 in GBM, p = 0.0016, (**Figure 3D**). Independently evaluating each subset of CD45-positive tumor-infiltrating immune cell groups, all showed greater than 50% of each cell type (B, T, NK, Mac/Mono/Gr, DCs) expressed PDL1, with approximately two-thirds of the dominant Mac/Mono/Gr population and virtually all (greater than 90%) T cells, B cells, DCs and NK cell population expressing PDL1 (**Figure 3E**). The high proportions of PDL1-expressing cells we find in the GBM immunome suggest these may play an important role in TME-mediated immunosuppression and effector T cell dysregulation, potentially contributing to the lack of durable response to CAR T therapy (37, 38). Conversely, examining PD1 expression on patient GBM immune infiltrates, the most numerous cells, including Mac/Mono/Gr (CD11b+), CD4+ T cells, and NK cells, had relatively few cells expressing PD1; however, although present in only small numbers, nearly all B cells and DC expressed PD1 (**Supplementary Figure S3D**). Additionally, although CD8 T cells were mostly absent in the GBM tumor microenvironment, those present all expressed both PD1 and PDL1 (**Figures 3E**, **Supplementary Figure S3D**). This profile suggests that B cells, DCs, and CD8 T cells could play a vital role in the naturally occurring GBM immune response.

Following up on earlier works demonstrating tumor-reactive CD8 T cells only expressed PD1 in the TME (39), we investigated PD1 and PDL1 expression in PBMC from both normal donors and GBM patients to compare with the TME infiltrates (**Supplementary Figures S3F, H**). PBMC from GBM patients contained fewer T cells (approx. 20% versus 90%), and more Mac/Mono/Gr, DCs, and B cells overall than PBMC from normal donors (**Supplementary Figure S3B**). The proportions of these cells that expressed PDL1 were similar, or less, in GBM patients’ periphery than those in normal donors, and were not reflective of the high levels of PDL1 expressed by immune cells in the GBM TME (**Figure 3E**, **Supplementary Figures S3E, F**). This differential PDL1 expression suggests localized upregulation within the TME whether by intrinsic or extrinsic factors. Evaluating PD1 in the periphery, the majority of all cell types in normal donor PBMC were PD1-negative, while those from GBM patients showed one-third or more of circulating Mac/Mono/Gr, DCs, and B cells expressed PD1 (**Supplementary Figures S3F–H**). In circulating T cells, the proportion expressing PD1 was similar between GBM patients and normal donors; however in total number there were fewer T cells overall in patients. This low circulating number of PD1 T cells in GBM patients could reflect the low tumor mutation burden generally found in GBM and suggests that T cells may play a minor role in patients’ endogenous GBM immune response (40–45).

The GBM samples evaluated here were all from newly diagnosed or recurrent tumor resections from untreated patients or patients treated with standard of care (chemotherapy, radiation, and resection), and none had received adoptive CAR T cell therapy. In agreement with prior published data, the patient GBMs we evaluated had very few CD8+ T cell infiltrates, aligned with the cold tumor phenotype typically associated with low tumor mutational burden (40–45). Treating a GBM patient with tumor-specific CAR T presumably alters the GBM TME in several ways. The mechanism of action upon tumor–CAR T cell interaction involves the scFv expressed by the T cell binding its cognate antigen, triggering T cell activation through signals from the co-stimulatory motif and the CD3ζ domain. This results in the upregulation of T cell activation markers, including CD69 and PD1, induction of the perforin-granzyme lytic pathway, and secretion of Type 1 immune cytokines, including tumor necrosis factor-alpha (TNFα), the lymphocyte pro-survival factor interleukin-2 (IL-2), and IFNγ, a pro-inflammatory cytokine. IFNγ in particular, induces pleiotropic effects in surrounding cells and is known to upregulate tumor cell antigen processing and presentation while paradoxically increasing tumor immune suppressive signals, including PDL1 (46). As a result, it is reasonable to consider GBM patient tumors treated with CAR T may respond to CAR T therapy by adaptively producing an even more immune-suppressive TME, specifically via upregulating the PD1-PDL1 axis and driving T cell exhaustion in response to IFNγ produced by the CAR T cells.

### Combination of anti–PD1 Ab with CAR T fails to enhance efficacy in an orthotopic GBM model

**Figure 4:**
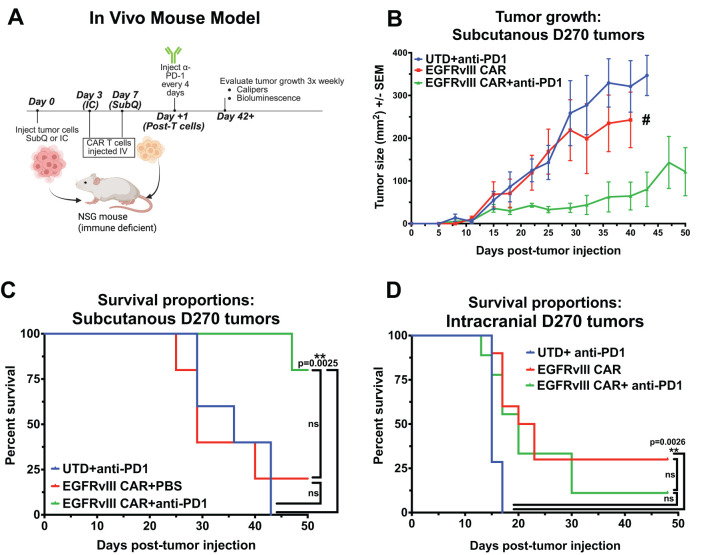
Combining EGFRvIII CAR T with anti-PD1 Ab improves CAR treatment of subcutaneously-implanted GBM xenografts in NSG mice, but has no impact in an orthotopic model. **(A)** Representative image illustrating the experimental setup for CAR T plus anti-PD1 Ab treatment in an established human GBM xenograft model in immune-compromised NSG mice, Created in BioRender. Cook, D. (2025) https://BioRender.com/j1brg3l. **(B)** Caliper-based tumor measurements (L x W) over 50 days in mice injected subcutaneously with D270 human GBM xenograft, and treated with T cells 7 days later. Treatment with anti-PD1 or PBS began on day 8 and continued every 4 days. Groups: untransduced T cells (UTD) plus anti-PD1 Ab, T cells expressing EGFRvIII-specific CAR, or T cells expressing EGFRvIII-specific CAR combined with anti-PD1 Ab treatment (n = 5 per group), (# single surviving animal). The mice where euthanized when tumor the tumor was 2 cm in any direction. **(C)** Kaplan-Meier survival curves for mice with subcutaneous D270 tumors receiving treatments as outlined in **(B, D)** Kaplan-Meier survival curves for mice with orthotopically injected intracranial D270 tumors, treated with UTD plus anti-PD1 Ab, EGFRvIII-specific CAR T cells, or EGFRvIII-specific CAR T cells plus anti-PD1 Ab (n = 7 per group). T cells were dosed i.v. 3 days post-tumor, and treatment with anti-PD1 Ab started one day post CAR T and was given i.p. every 4 days. Mice were euthanized when they met predefined humane endpoint criteria, including ≥20% loss of original body weight, Body Condition Score (BCS) below 2, or loss of normal physiological function (e.g., impaired mobility, grooming, or feeding). Statistical analyses were performed using the Log-rank (Mantel-Cox) test and statistical significance was denoted as follows: P < 0.01 (**), and P ≥ 0.05 was considered not significant (ns).

To generate a PD1-PDL1 immunosuppressive CAR T treatment model of GBM *in vivo*, we used the human GBM xenograft D270 with endogenous EGFRvIII expression, and demonstrated *in vitro* upregulation of PDL1 upon exposure to IFNγ (**Supplementary Figure S4A**). For treatment, we used our previously published second-generation EGFRvIII CAR T (co-stimulated with 4-1BB and activation through CD3ζ) in both subcutaneous and orthotopic brain tumor xenografts in NSG mice (**Figure 4A**) (6, 47, 48). We initially evaluated the impact of PD1-PDL1 CPI on tumors located outside the brain. D270 GBM were implanted subcutaneously on the flank of NSG mice 7 days before treatment with sub-optimal numbers of i.v. injected untransduced T cells (UTD) or EGFRvIII CAR T cells. Anti-PD1 Ab or PBS followed this CAR T treatment, injected i.p., starting one day after T cell infusion and repeating every four days. We found that this dosage of EGFRvIII CAR T alone had a modest impact on tumor size compared with PD1 blocking Ab treated UTD T cells, and conferred no improvement in overall survival (OS) per Kaplan-Meyer curve (median OS = 36d for UTD + PD1 Ab, 28d for CAR T alone). However, mice treated with CAR T cells combined with anti–PD1 Ab exhibited significantly smaller tumors and improved survival compared to CAR T cells alone (p = 0.0025, Log-rank [Mantel-Cox] test), with median OS not reached by 50 days (**Figures 4B, C**).

Since tumors located in the periphery are readily accessed by soluble factors in the blood, including Abs, this subcutaneous model does not reproduce the TME in GBM, with restricted access to the brain. To test whether the BBB is a factor in treatment with CPI Abs, we repeated this GBM xenograft model, treating with CAR T both with and without i.p. delivered soluble anti-PD1 Abs in the same model, but with an orthotopic tumor implanted in the brain. Upon treatment, the addition of anti-PD1 Ab to EGFRvIII CAR T had no impact, with statistically similar OS by Kaplan-Meyer graph. Median OS is 15d for negative control UTD T cells plus PD1 Ab and 21.5d for EGFRvIII CAR T alone, with addition of CPI Ab resulting in 20d OS for CAR T plus PD1 Ab (**Figure 4D**). Long-term animal survival at the end of the experiment on d48 were 0% (UTD + Ab), 30% (CAR T), and 11% (CAR T + Ab). Descriptions of the BBB excluding passage of circulating Abs into the CNS, have been well described (49, 50). Therefore, it is unsurprising that soluble injected anti-PD1 Ab does not augment CAR T treatment of brain tumor progression in the orthotopic model. Collectively, these data suggests that the PD1-PDL1 axis can inhibit CAR T cell function in GBM treatment *in vivo*, and PD1 blockade with anti-PD1 Abs can overcome that inhibition in peripheral disease. However, systemic administration of anti-PD1 Abs does not enhance therapeutic efficacy against CNS-localized GBM, potentially due to the BBB preventing Abs from entering the CNS, thus preventing Abs engagement with CAR T cells at the tumor site.

### Antibody receptor modified ARMed CAR T cells can secrete anti-PD1 Abs/minibodies

To overcome the access barrier of the BBB in delivering CPI Abs to disrupt the PD1-PDL1 axis on CAR T cell function in orthotopic GBM, we further genetically modified the EGFRvIII CAR T cells to perform a second function, specifically to act as mini bio-factories, producing their own anti-PD1 Ab for CPI. To first determine function, we utilized an *in vitro* cellular system to quantitatively establish PD1-PDL1 binding and functional readout on CAR T cell activation and subsequent inhibition (**Supplementary Figure S4B**). This model used K562 as target cells constitutively expressing CD19 (a surrogate tumor antigen), and PDL1, or K562 negative control target cells that express neither CD19 nor PDL1 (**Supplementary Figure S4C**). We used second-generation CD19 CAR T (with 4-1BB and CD3ζ) as effector cells. To determine the impact of PD1-PDL1 binding on antigen-specific CAR T cell activation, we overexpressed PD1 via mRNA electroporation of CAR T cells (**Supplementary Figure S4D**) prior to co-culture. Following co-culture of effector CAR T cells with K562 targets, T cell activation was assessed by intracellular cytokine staining and flow cytometric analysis. We found that overexpression of PD1 on CAR T cells significantly reduced CAR T cell activation, as measured by production of IFNγ (p = 0.0024), IL-2 (p = 0.0173), and TNFα (p = 0.0002), compared with PD1-negative CAR T cells (**Supplementary Figures S4E–G**). We also evaluated T cell proliferation by measuring CFSE dilution and found that CD19 CAR T cells co-cultured with K562 expressing CD19 and PDL1 resulted in the proliferation of CD19-specific CAR T cells, but this antigen-specific proliferation was significantly reduced when the CD19 CAR T cells also overexpressed PD1 (p = 0.02) (**Supplementary Figure S4H**). These data support that PD1–PDL1 interactions inhibit the function of antigen-specific CAR T cells. Together with our *in vivo* results using PD1 Ab and CAR T cell combination therapy, this suggests that the PD1–PDL1 signaling axis may contribute to immune suppression in patients undergoing clinical CAR T cell treatment (17–19).

**Figure 5:**
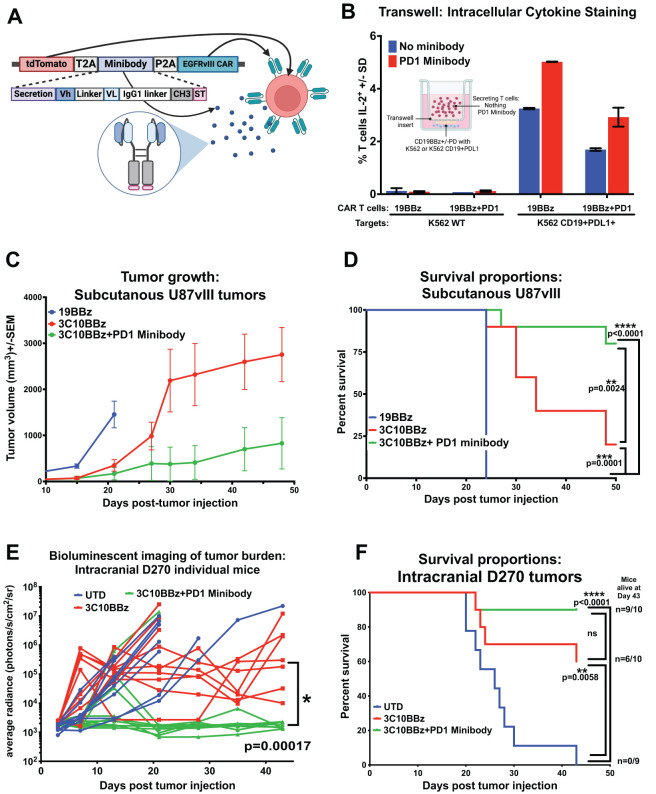
Antibody Receptor Modified (ARMed) CAR T secreting anti-PD1 mini-Ab can rescue CAR T function in orthotopic GBM xenograft murine model. **(A)** Schematic representation of the cDNA and final protein structure of EGFRvIII CAR T engineered to produce and secrete an anti-PD1 minibody, created in BioRender. Cook, D. (2025) https://BioRender.com/l51h190. **(B)** Bar graph of intracellular cytokine detection of IL-2 by flow cytometry of a transwell assay, where the T cells producing the PD1 minibody were not in direct contact with the tumor cell targets. Transwell image Created in BioRender. Cook, D. (2025) https://BioRender.com/2j7t6i8 **(C–F)** NSG mice were injected subcutaneously (subQ) or orthotopically intracranially (IC) with GBM xenografts and treated with engineered T cells either 7 days post-tumor injection for subQ or 3 days later in the IC model. **(C)** Tumor volume (0.5xLxW^2^) measurements were taken with calipers over time in mice injected subcutaneously with U87 EGFRvIII-positive human GBM cells (U87vIII) and treated with negative control CAR T cells (CD19BBζ), EGFRvIII-specific CAR T cells (3C10BBζ), or EGFRvIII-specific ARMed CAR T cells secreting the anti-PD1 minibody (3C10BBζ + PD1 minibody) (n = 10 per group, except the UTD group (n = 9), where one mouse did not develop a tumor). **(D)** Kaplan-Meier survival curves for mice in **(C)** with subcutaneous U87vIII tumors. **(E, F)** To follow tumor size and overall survival, the D270 GBM was stably transduced with luciferase, average bioluminescence intensity (BLI) tracking over time for each treatment group after injection of luciferin-D substrate provided i.p. Mice injected intracranially with D270 GBM and treated i.v. with: untransduced T cells (UTD), EGFRvIII-specific CAR T cells (3C10BBζ), or ARMed EGFRvIII-specific CAR T cells secreting the anti-PD1 minibody (3C10BBζ + PD1 minibody), show **(E)** tumor burden by BLI, and **(F)** overall survival by Kaplan-Meier survival curves (n=10 per group). Mice were euthanized when they met predefined humane endpoint criteria, including ≥20% loss of original body weight, Body Condition Score (BCS) below 2, or loss of normal physiological function (e.g., impaired mobility, grooming, or feeding). Statistical analyses were conducted using the Log-rank (Mantel-Cox) test. Statistical significance was denoted as follows: P < 0.05 (*), P < 0.01 (**), P < 0.001 (***), and P < 0.0001 (****); P ≥ 0.05 was considered not significant (ns).

To address the limitations of systemic Ab access to the brain, we utilized CAR T cells, shown to readily traffic across the BBB to gain access into the brain, as a natural biologic delivery mechanism, targeting T cells to specific antigens within the brain, in this case, EGFRvIII on GBM, and there to produce and secrete anti-PD1 Ab minibodies *in situ* (6). These Ab receptor-modified (ARMed) CAR T cells can provide PD1 CPI *in cis*, or to provide CPI for other infiltrating anti-tumor immune cells close proximity *in trans*. **Figure 5A** depicts the minibody CAR T plasmid design we used in pTRPE lentiviral vector to transduce human T cells. To detect the minibody, we added a small epitope tag (StrepTagII) attached to the c-terminus, previously used on secreted proteins and single-chain variable fragments (scFv) in CAR T cells (51, 52). This tag allowed us to collect ARMed CAR cell supernatant and enrich for anti-PD1 minibody protein by column purification and run on SDS PAGE gel, followed by western blot detection using a StrepTag Ab (**Supplementary Figure S5A**). We show one specific product eluted from the column at the predicted size of 45 kDa as a monomer in reducing conditions. To directly detect the secreted PD1 minibody functional binding to PD1, we developed an ELISA using immobilized recombinant PD1 protein and anti-human IgG detection mAb that directly measures anti-PD1 minibody. We demonstrate direct and specific detection of the PD1 minibody in ARMed CAR T supernatant (**Supplementary Figures S5A, B**). We evaluated secreted minibody functionality *in vitro* based on expected signaling inputs shown in **Supplementary Figure S4B**. We confirmed that anti-PD1 ARMed antigen-specific CAR T can block PD1-PDL1 inhibition *in trans* using a transwell system, where the minibody-secreting cells are not in direct contact with the effector and target cells. CD19 CAR T with or without mRNA electroporated PD1 expression, cultured with CD19 PDL1 positive K562 targets, demonstrated antigen-specific IL-2 production was markedly reduced when CAR T overexpressed PD1 but could be rescued when ARMed T cells secreting anti-PD1 minibody were present *in trans* (**Figure 5B**).

### PD1 ARMed CAR T cells have enhanced anti-tumor function *in vivo*

To determine whether the PD1 minibodies secreted by ARMed EGFRvIII CAR T cells function *in vivo* to rescue PD1-PDL1 inhibited T cell function and improve CAR T therapy, we repeated the previous *in vivo* NSG xenograft flank model of GBM, this time using anti-PD1 ARMed EGFRvIII CAR T, EGFRvIII CAR T alone, or negative control CD19 CAR T cells to treat U87vIII GBM, naturally expressing high levels of PDL1, which further increased upon exposure to IFNγ (**Supplementary Figure S4A**). Tumors were implanted subcutaneously on the rear flank, and T cells were injected i.v. 7 days later. The anti-tumor impact was evaluated by measuring tumor size via calipers, and evaluating overall survival (**Figures 5C, D**). All mice treated with negative control CD19 CAR T reached the predetermined experimental endpoint by tumor size or animal morbidity 24 days (median OS = 24d) after tumor engraftment. EGFRvIII CAR T treated mice showed a significant reduction in tumor size compared with negative controls, with a median OS of 34 days and 10% of mice surviving to 50 days (end of the study). ARMed EGFRvIII CAR T cells secreting PD1 minibody further augmented tumor control, with significantly increased overall survival compared to CAR T alone, median OS not reached at 50d, 80% of mice surviving to end of study in this subcutaneous GBM model.

Next, we wanted to evaluate whether PD1 ARMed CAR T cells could overcome the limitations of CNS-localized tumors and could outperform CAR T cells in the D270 orthotopic GBM xenograft NSG mouse model, where combination with anti-PD1 Ab therapy failed. We injected D270 GBM intracranially in NSG mice and confirmed tumor engraftment with bioluminescent imaging (BLI). Three days later, mice were i.v. injected with 5 million EGFRvIII CAR T cells, either ARMed with PD1 minibody or CAR T alone, or untransduced T cells (UTD) as negative controls. Mice treated with UTD showed rapid tumor growth and median OS of 26 days, with 0/9 (0%) mice surviving to the end of the study at day 43 (**Figures 5E, F**). The EGFRvIII CAR T alone treated mice showed reduced tumor BLI compared with UTD, with median OS not reached at 43d, and 6/10 (60%) mice surviving to the end of the study on day 43 (p = 0.006). Mice treated with PD1 ARMed EGFRvIII CAR T cells exhibited a significant reduction in tumor BLI compared with both UTD cells and CAR T cells alone (p = 0.00017 by multiple Mann-Whitney tests; **Figure 5F**). Additionally, ARMed CAR T mice did not reach median OS with 9/10 (90%) mice surviving through d43 endpoint. A limitation of CAR T xenograft mouse models is the potential for human T cells to recognize and eventually reject mouse host cells as foreign, leading to xenograft graft-versus-host disease (xGVHD). Although the severity and timing of xGVHD can vary depending on the T cell donor, it typically restricts the duration of these experiments to 6–12 weeks, often necessitating termination due to animal morbidity even in absence of tumor before reaching median overall survival (53). Although statistical significance was not reached for OS between ARMed CAR T versus CAR T alone treated mice (median OS was not reached for either), CAR T alone treated mice had 6/10 (60%) mice surviving through the end of the study, compared with 9/10 (90%) treated with ARMed CAR T (**Figure 5F**), suggesting a potential survival benefit. This benefit is supported with the corresponding BLI data, clearly demonstrating markedly reduced tumor burden in the ARMed CAR T group, compared with the CAR T alone (**Figure 5E**).

In peripheral blood samples from the orthotopic model, human T cells were detected at comparable levels in all groups at 18 days post-infusion (**Supplementary Figure S5C**). In both CAR T groups (ARMed and not), human T cells could still be detected 29 days post-infusion (**Supplementary Figure S5D**), demonstrating that CAR T cell persistence in the periphery is not sufficient to confer anti-tumor CAR T function in this model of PD1-PDL1 mediated immune suppression, and that T cell function against peripheral tumors could be improved by administration of CPI Abs. However, only ARMed CAR T cell secreting CPI Ab was sufficient to eliminate GBM in the CNS behind the BBB. Our data suggests that engineering ARMed CAR T cells to secrete checkpoint Abs could overcome checkpoint-induced treatment barriers for solid tumors, improving clinical outcomes, particularly in barrier-protected organs, such as GBM.

In summary, understanding the composition and phenotype of immune infiltrates and the potential role of PD1-PDL1 mediated immunosuppression in GBM is essential for developing effective immunotherapeutic strategies. Here, we have shown evidence from experimental murine GBM models and patient GBM samples highlighting the role of infiltrating immune cells and their potential contributions to checkpoint PD1-PDL1 induced immune suppression in GBM. We explored the combination of treatment with CAR T plus PD1 Abs, focusing on the challenges associated with blocked access of systemically delivered Abs across the BBB into the brain in orthotopic GBM models. We developed a GBM-targeted 4^th^ generation ARMed CAR T with the capacity to generate and secrete anti-PD1 checkpoint blockade Abs as minibodies. We demonstrated that PD1 minibodies secreted by these ARMed CAR T were detectable and functional in cellular assays *in vitro*. Finally, we demonstrated preclinical proof of concept of therapeutic efficacy of 4^th^ generation solid tumor targeting ARMed CAR T cells in a GBM mouse model in subcutaneous and orthotopic tumors. These data suggest that ARMed CAR T cells can cross the BBB into the brain and secrete anti-PD1 minibodies as a potential dual-action therapeutic strategy for treating GBM. This therapy holds promise for meaningful clinical benefit and improved outcomes for patients with GBM and other brain diseases.

## Discussion

Glioblastoma (GBM), the most aggressive primary brain tumor in adults, presents a formidable challenge to effective treatment due to its infiltrative nature, low tumor mutational burden, and complex immunosuppressive microenvironment. Understanding the composition and dynamics of immune cell infiltration within the tumor microenvironment is crucial for developing effective immunotherapeutic strategies. In this study, we evaluated immune infiltration in an immunocompetent syngeneic murine GBM model and compared it with freshly resected and archival GBM patient samples. In both models, we observed abundant immune infiltrates, predominantly composed of Mac/Mono/Gr cells, along with smaller populations of DCs, B cells, T cells, and NK cells. These findings are consistent with previous reports demonstrating that tumor-associated macrophages (TAMs), including infiltrating macrophages, MDSCs, and resident microglia, constitute a major component of the GBM microenvironment in both mice and humans (54, 55). Although not detected in normal brain tissue, we cannot exclude the possibility that the observed increase in Mac/Mono/Gr cells reflects activated microglia in response to the tumor, rather than newly infiltrating immune cells. Recent multispectral imaging analyses in human GBM have also revealed that macrophages are the most abundant immune population, followed by T cells and neutrophils, with NK and NKT cells being comparatively rare, and identified novel immunosuppressive subsets such as PD1+ NK cells, PD1+CD8+ T cells, and PDL1+ neutrophils (54, 55). Single-cell RNA sequencing studies in mice and humans have further underscored the critical role of myeloid cells, including microglia, macrophages, and MDSCs, in shaping the immunosuppressive landscape of GBM (56–58). Additionally, recent work identified distinct MDSC populations within GBM that engage in cross-talk with tumor cells to promote tumor growth and immune evasion, emphasizing the complexity and central role of myeloid-driven immunoregulation in GBM (58).

In our murine GBM model, we observed that immune cells were significantly reduced in the spleen during tumor progression. This finding aligns with previous work by Chongsathidkiet et al., who reported that patients with GBM had significantly smaller spleen volumes compared to age-matched controls, a result that was also validated in two independent mouse models of GBM (59). Consistent with these systemic immune alterations, we found that GBM patients exhibited significantly reduced numbers of lymphocytes in their PBMCs compared to healthy donors (59–62). Prior studies have proposed multiple mechanisms for this lymphopenia, including sequestration of T cells within the bone marrow and T cell apoptosis driven by soluble factors in the tumor microenvironment (59, 62). In addition, our study demonstrated that MHC-II cells that had phagocytosed tumor-derived GFP could be found in the draining lymph nodes. This is consistent with recent reports highlighting the role of dendritic cells in acquiring tumor antigens within the brain and migrating to cervical lymph nodes in GBM models (63). Together, these findings underscore a coordinated disruption and redistribution of immune cells in both the systemic and CNS compartments during GBM progression.

A notable difference between the murine and patient GBM was the degree of T cell infiltration into the tumor. There was a high proportion of CD4+ and CD8+ T cells in the GL261 mouse GBM model, notably absent in GBM patient samples. While patient GBMs did contain a subset of T cells, these were not a predominant population and were almost entirely composed of CD4 T cells. The GL261 C57Bl/6 mouse model has been reported to have a strong T cell infiltration not generally observed in human GBM, presumably due to its high tumor mutational load (29, 30, 41, 64). Whole-genome sequencing and RNA sequencing of GL261 has shown these tumor cells have a high mutational burden, and express close to 5,000 non-synonymous exome mutations, which is significantly higher than typically found in patients’ GBM (29, 43). Furthermore, it has been demonstrated that these mutations lead to the expression of neoepitopes that can be recognized by T cells. This may explain the high T cell infiltration we observed in this model (29). Despite these differences, murine models have been crucial in advancing GBM CAR T cell therapy from preclinical research to clinical applications for patients (6, 12, 13, 23, 48, 65).

Our study found that high proportions of all immune subsets in the patient GBM immune infiltrates had PDL1 expression, and many of the non-immune (tumor) cells also expressed PDL1. We confirmed this finding by IHC of archived FFPE tumor sections from both newly diagnosed and matched recurrent GBM patients, where the majority of samples (80%) showed PDL1 staining, some with focal staining consistent with site-specific upregulation in response to IFNγ produced by infiltrating immune cells. PDL1 expression on immune cells in GBM has not been thoroughly investigated. However, a recent study showed that across multiple cancers, responses to anti-PDL1 antibody therapy were observed in individuals expressing high levels of PDL1, especially on tumor-infiltrating lymphocytes, suggesting a functional role of PDL1 on immune cells (66). The extent of PDL1 displayed by both tumor and immune infiltrates in these patient resections suggests that PD1-PDL1 may be a significant mechanism of immune evasion and T cell suppression in GBM, as supported by other studies (67, 68). It has previously been shown that PDL1 expression on infiltrating immune cells in lung cancer is highly associated with the presence of M2-polarized macrophages, consistent with our findings in fresh and archival samples of GBM (69). This concept is further supported by a previous clinical trial administering EGFRvIII CAR T for GBM. Specifically, tumor samples taken before and after CAR T treatment showed multiple biomarkers of adaptive tumor response to T cell-mediated anti-tumor activity, including upregulation of PDL1 and other markers associated with suppression of the immune response (6). One major limitation to our human GBM flow cytometry analysis was due to the low frequency of immune infiltrates, which limited us to identifying only significant immune populations, and more detailed phenotypic or functional characterization was not feasible. These limitations should be considered when interpreting the scope of our human immune profiling results.

In our experimental CAR T treatment of GBM xenograft NSG mouse model, we demonstrated that administering a suboptimal number of EGFRvIII CAR T cells had minimal impact on established subcutaneously implanted PDL1–positive D270 GBM tumors. Regular systemic injections of anti–PD1 Abs combined with the CAR treatment significantly improved anti-tumor efficacy in these peripheral tumors. However, when the same tumor was implanted orthotopically within the brain, the combination treatment of anti–PD1 Ab and CAR T cells did not enhance therapeutic response compared to CAR T cell therapy alone. This suggests that PDL1–mediated immune suppression of CAR T cell function occurs *in vivo* and can be successfully treated with anti–PD1 CPI Abs in peripherally located tumors; but, since Abs cannot effectively cross the BBB, there is no rescue of CAR T cell activity against tumors within the brain. This data is unsurprising, as it is a known problem in treating patients with brain diseases that Abs and many other therapeutic agents cannot cross the BBB to reach sites within the brain. In contrast to circulating Abs, CAR T cells can readily traverse from the circulation across the BBB and access brain tumors. Clinical and preclinical studies have shown that CAR T cells can penetrate the CNS, localize to tumor sites, and exert anti-tumor effects (6, 26, 70). Despite initial anti-tumor activity observed in recent clinical trials, CAR T cell therapy has failed to reproducibly provide a prolonged clinical benefit, mainly attributed to T cell functional exhaustion, typically characterized by high levels of PD1 and other immunosuppressive markers. This suggests that strategies to target PD1-PDL1 axis may overcome the immunosuppressive impact of the tumor microenvironment and enhance the durability of CAR T cell therapeutic function in GBM (6, 11, 13). Based on these findings, a follow-up clinical trial combined EGFRvIII-targeted CAR T cells with anti–PD1 checkpoint Ab administration for treating GBM (26). Unfortunately, the results were disappointing, showing no improvement over the CAR T alone trial, and no increase in clinical anti-tumor response (71, 72). This failure underscores the need for novel strategies to overcome the immunosuppressive tumor microenvironment and enhance the durability of CAR T cell therapies for patients with GBM.

To address these challenges, we engineered CAR T cells to secrete anti–PD1 minibodies, creating ARMed CAR T cells that serve as living bio-factories and drug delivery systems. This approach leverages synthetic biology to modify CAR T cells to localize to tumor antigens and secrete CPI locally within the TME. Minibodies are Ab fragments comprising a single chain variable fragment (scFv) fused to a CH3 domain that dimerize once the protein is made. This results in a molecule much smaller than a full Ab but remains bivalent. Minibodies are advantageous due to their increased stability and prolonged half-life compared to scFv and diabodies (scFv^2^) (73–76). We have previously shown the ability to reprogram IL13Rα2 CAR T cells to secrete checkpoint blockade minibodies and that these can augment CAR T treatment in subcutaneously implanted murine GBM xenograft models, with results similar to CAR T and checkpoint Ab combination therapy (77). In orthotopic GBM, our approach harnesses the inherent CAR T cells’ ability to infiltrate the CNS, and enhances the efficacy of CAR T cells while minimizing systemic exposure to checkpoint inhibitors, potentially reducing co-incident toxicities. Here, we have shown our ARMed CAR T cells demonstrated enhanced anti-tumor activity in subcutaneous and intracranial GBM models, including the ability to cross the BBB and function within the brain. Secreting anti–PD1 minibodies directly at the tumor site protected the CAR T from the immunosuppressive signals in the GBM TME, improved T cell persistence and anti-tumor function (**Supplementary Figure S5E**). This 2-in-1 strategy overcomes the limitations of deliveriing systemic Abs across the BBB, allowing for therapeutic levels of CPI Abs to accumulate locally and treat brain tumors.

Here, we show that ARMed CAR T engineered to secrete anti-PD1 minibodies can improve anti-tumor function not only in systemically located tumors but also deliver sufficient Ab levels within the brain at the site of the tumor to rescue CAR T function in an orthotopic D270 xenograft CAR T-resistant mouse model. We acknowledge that a limitation of the *in vivo* experiments is the absence of certain control groups, such as direct comparisons between ARMed CAR T cells and combination CAR T plus anti–PD1 Ab therapy, as these controls were used in separate experiments. Cumulatively these experiments demonstrate the potential to directly translate into clinical trials for patients with GBM and other disease located within the brain behind the BBB. As CAR T cells accumulate at the tumor site, the effective checkpoint blockade Ab they serete can increase localized concentration *in situ*, in contrast with barrier-restricted entry to systemically delivered Abs, or even intracerebrally delivered Abs, which are actively pumped out by transporters like the neonatal Fc receptor (FcRn) located in the BBB. Also, unlike Abs, which have a limited persistence/half-life, T cells can potentially remain over the patient’s lifespan. This *in vivo* continued supply of Abs can remain relatively constant for as long as the tumor persists, offering the possibility of durable long-term treatment without repeated administrations. In the absence of tumor, directed ARMed CAR T cell populations can contract and disperse through the circulation, reducing any site-specific CPI Ab concentrations below functional levels.

Recently, a publication by Simic et al. further developed the concept of controlled engineering of T cells to act as mini bio-factories *in vivo*, demonstrating the potential for these cells to produce not only Abs but potentially any biologic drug, including immune- or tumor-modulating cytokines or peptides to modify the TME (78). Importantly, they demonstrate the ability to turn on and off these functions using logic-gated genetic switches, including ‘AND’ and ‘OR’ gates, for example, turning on protein secretion only when in the presence of an organ-specific marker or in a specific microenvironment. In conclusion, these works demonstrate that engineering CAR T cells to secrete anti–PD1 Abs/minibodies offers a promising solution to the challenges of treating patients with GBM, overcoming dual challenges of targeting specificity (EGFRvIII or other tumor-associated antigen), and overriding PD1-PDL1 induced immunosuppression encountered in the TME, whether from tumor or other immune-infiltrating cells. This innovative approach combines the benefits of directed cellular therapy with localized Ab-based immunomodulation, overcoming the limitations of drug delivery across the BBB and enhancing anti-tumor responses. The success of ARMed CAR T cells in preclinical models supports their translation into clinical trials that could significantly improve outcomes for patients with GBM and other high-unmet medical need CNS diseases. Future developments may include incorporating safety mechanisms like a ‘suicide switch’ such as CD20, that could be added to the ARMed CAR T for rapid elimination via treatment with drugs like Rituximab; alternatively, an ‘on-switch’ for the minibody could be added, driven by T cell-intrinsic activation factors on an NFAT- or other site-specific promoter, thus controlling not only the localization of the secreted Abs, but their timing also, adding a further potential measure of safety. Undoubtedly, we will learn more about the capabilities and limitations of these types of ARMed CAR T cell therapies following their use in early-phase clinical trials.

## Materials and methods

### Fresh brain tumor digestion and immune cell collection

Primary GBM cell lines were derived directly from patients undergoing neurosurgical resection at the Hospital of the University of Pennsylvania, following their Institutional Review Board-approved protocol. Written consent was obtained from all patients following the protocol. Freshly resected glioma tumor samples were enzymatically dissociated into single cells using the Papain Dissociation System (Worthington Biochemical Corporation, Cat#:LK003160). Then, CD45 microbeads (Miltenyi Biotech, Cat#130-045-801, RRID: AB_2783001) isolated immune cells from the dissociated tumor by positive selection. Enrichment was assessed by Ab staining and flow cytometry in both populations for CD45 AlexaFluor647 (BioLegend, Cat#:304020, RRID: AB_493034). The CD45-positive cells were cryopreserved for later analysis.

### Immunohistochemistry of human GBM samples

GBM sections were obtained from samples from the University of Pennsylvania Brain Tumor Tissue Bank. IHC of formalin-fixed and paraffin-embedded tissues was performed on a Leica Bond instrument using the Bond Polymer Refine Detection System using the following Abs: CD3 (Leica, Cat#:PA0553-U), CD33, HLA-DR, CD11c, and PDL1. Heat-induced epitope retrieval was required and done for 20 min with ER1 solution (Leica Microsystems). Tonsil tissue was also included as an immune tissue control.

### Immunohistochemistry of murine samples

Mouse tumor tissues were formalin-fixed in 10% neutral buffered formalin (NBF) at ten times the tissue volume for 24–48 hours. The fixed samples were sent to the Pathology core for paraffin embedding and sectioning. Sections in FFPE were stained using F4/80 Ab (Thermo Fisher Scientific, Cat#:14-4801-82, RRID: AB 467558) or anti-EGFRvIII Ab (3C10) on glass microscope slides. Slides were deparaffinized in xylene (2 changes, 5 min each) and rehydrated through graded ethanol. Endogenous peroxidase was blocked using 0.3% H_2_O_2_/methanol for 30 min. Antigen retrieval was performed using pH 9 Retrieval Solution (DAKO, Cat#:S236784-2) in a pressure cooker. After cooling, slides were rinsed in 0.1 M Tris buffer and blocked with 2% fetal bovine serum for 10 min, followed by avidin/biotin blocking (Vector Laboratories, SP-2001). The slides were incubated overnight at 4°C with F4/80 Ab (1:100 dilution) or EGFRvIII Ab (1:100 dilution), followed by a 30-minute incubation with biotinylated anti-Rat IgG or anti-Rabbit IgG (1:200 dilution, Vector Laboratories) at room temperature. Slides were treated with the avidin-biotin complex (Vector Laboratories) for 30 min, developed using DAB (DAKO Cytomation) for 10 min, counterstained with Hematoxylin (30 sec), and dehydrated through graded ethanol and xylene before coverslipping.

### PD1 minibody detection *in vitro*

Co-culture supernatant was concentrated using Strep-Tactin XT 4Flow (iba, Cat#:2-5998-000) to isolate strep-tagged PD1 minibody. Multiple fractions were isolated and separated on 4–12% Bis-Tris gels (Thermo Fisher Scientific, Cat#:NP0321BOX) and transferred to nitrocellulose membranes (Thermo Fisher Scientific, LC2001). Membranes were blocked with Odyssey Blocking Buffer (Li-Cor, Cat#:927-40000). Step-Tag minibody protein was detected using Strep-MAB classic (iba, Cat#:2-1509-001, RRID: AB_3095590) and IRDye 800CW Donkey anti-Mouse (Li-Cor, Cat#:926-68171). Strep-tagged PD1 minibody was also detected by ELISA using the DuoSet ELISA ancillary reagent kit 2 (R&D Systems, Cat#:DY008B). Briefly, a 96-well plate was coated with PD1 (Abcam, Cat#:ab174035) overnight at room temperature. The next day, supernatants from T cells transduced with Strep-tagged PD1 minibody or untransduced T cells were either unconcentrated or concentrated using Strep-Tactin XT 4Flow was added to the PD1 pre-coated plate and incubated for two hours at room temperature. For detection, goat anti-human IgG HRP was read on a plate reader OD 450 nm.

### CAR T expression

To detect CAR expression, cells were stained using goat-anti-human F(ab’)-biotin (Jackson ImmunoResearch, Cat#:109-065-097, RRID: AB_2337629) in PBS containing 0.2% bovine serum albumin at 1 × 10^6^ cells/ml (FACS buffer) and incubated at 4°C for 20 min. After incubation cells were washed with FACS buffer, and secondary detection was carried out by adding streptavidin-coupled PE (BD Pharmingen, Cat#:554061, RRID: AB_10053328) incubated at 4°C for 30 min. After incubation, cells were washed twice and resuspended in FACS buffer. Fluorescence was assessed using a BD LSR II flow cytometer, and data were analyzed with FlowJo software.

### Tumor cell PDL1 expression

Tumor cells were cultured in the absence or presence of IFNɣ (R&D systems, Cat#:285-IF-100) at 20 ng/mL, 37°C in a 5% CO_2_ incubator for 24 hrs before being harvested for staining with PDL1(BioLegend, Cat#:329706, RRID: AB_940368). K562 cells expressing both PDL1 and CD19 were stained for PDL1 (BioLegend, Cat#:329706, RRID: AB_940368) and CD19 Abs (BioLegend, Cat#:302215, RRID: AB_314245) for cells expressing CD19.

### Intracellular cytokine analysis

CAR-transduced or untransduced T cells were co-cultured with target cells (tumors, cell lines, or human primary cells) in a 1:1 ratio at 2 × 10^6^/mL in 96-well round bottom tissue culture plates at 37°C, 5% CO_2_ for 6 hours in RPMI 1640 plus 10% FBS in the presence of Golgi inhibitors monensin (BD GolgiStop, Cat#:51-2092KZ) and brefeldin A (BD GolgiPlug, Cat#:51-2301KZ). Cells were washed, stained with live/dead viability stain (Thermo Fisher Scientific, L34955), followed by surface staining for CD3 (Thermo Fisher Scientific, Cat#:17-0037-42, RRID: AB_1907372), then fixed and permeabilized (Thermo Fisher Scientific, GAS001/2S100), and intracellularly stained for IFNɣ (BioLegend, Cat#:506507, RRID: AB_315440), TNFα (BioLegend, Cat#:502938, RRID: AB_2562741), and IL-2 (Thermo Fisher Scientific, Cat#:46-7029-42, RRID: AB_1834419).

### CFSE proliferation

T cells that were electroporated with *in vitro* transcribed mRNA 24hrs prior were harvested and labeled with 5 mM carboxyfluorescein diacetate succinimidyl ester (CFSE) (Thermo Fisher Scientific, Cat#:C34554), then plated in a 96-well plate at 1:1 ratio T cells to tumors (K562 PDL1/CD19), and incubated at 37°C, 5% CO_2_ in RPMI 1640 plus 10% FBS, with analysis by flow cytometry at day 2, day 4, day 8. All cells were harvested and stained with CD3 Ab to identify T cells and then gated on CFSE-positive to determine the proliferation of T cells.

### Murine lymphocyte panel

Isolated CD45-positive cells were stained with live/dead viability stain (Thermo Fisher Scientific, Cat#:L34955) for 30 minutes at room temperature. Then, the samples were stained with the following Abs: CD45 (BioLegend, Cat#:103136, RRID: AB_2562612), CD3e (BioLegend, Cat#:100310, RRID: AB_312675), CD8a (BioLegend, Cat#:100730, RRID: AB_493703), CD11c (BioLegend, Cat#:117308, RRID: AB_313777), CD11b (BioLegend, Cat#:101242, RRID: AB_2563310), Gr1 (BioLegend, Cat#:108424, RRID: AB_2137485), F4/80 (BioLegend, Cat#:123116, RRID: AB_893481), CD19 (BioLegend, 115543, RRID: AB_11218994), CCR7 (BioLegend, 120116, RRID: AB_2291144), CD49b (BioLegend, Cat#:108922, RRID: AB_2561460), pan MHC II (BioLegend, Cat#:107635, RRID: AB_2561397), CD206 (BioLegend, Cat#:141721, RRID: AB_2562340) and murine Fc block (e-Bioscience, Cat#:16-0161-81) in a master mix for 4 minutes before washing with FACS buffer (2 mM EDTA, 5% FBS, 1xPBS). Cells were then fixed using reagent A (Thermo Fisher Scientific, Cat#:GAS001).

### Human lymphocyte panel

Isolated CD45-positive cells were stained with live/dead viability stain (Thermo Fisher Scientific, Cat#:L34955) for thirty minutes at room temperature. Then, the samples were stained with the following Abs: CD45 (BioLegend, Cat#:304048, RRID: AB_2563129), CD3e (BioLegend, Cat#:300436, RRID: AB_2562124) CD8a (BD Biosciences, Cat#:561423, RRID: AB_10682894), CD4 (BioLegend, Cat#:317444, RRID: AB_2561866), CD11c (BioLegend, Cat#:301623, RRID: AB_10643589), CD11b (BioLegend, Cat#:101241, RRID: AB_11218791), CD19 (BioLegend, Cat#:302215, RRID: AB_314245), CD56 (BioLegend, Cat#:318333, RRID: AB_11142683), CD33 (BioLegend, Cat#:303303, RRID: AB_314343), PD1 (BioLegend, Cat#:329940, RRID: AB_2563659), PDL1 (BioLegend, Cat#:329706, RRID: AB_940368), CD206 (BD Biosciences, Cat#:551136, RRID: AB_394066) and Fc block (Thermo Fisher Scientific, Cat#:14-9161, RRID: AB_468581) in a master mix at 4°C for 30 minutes before washing with FACS buffer (2 mM EDTA, 5% FBS, 1xPBS). Cells were then fixed using reagent A (Thermo Fisher Scientific, Cat#:GAS001).

### Flow cytometry analysis

All flow cytometry samples were acquired on Beckton-Dickinson LSR II, and data were analyzed with FlowJo v8.8.7 (TreeStar).

### Generation of lentivirus

Lentiviral supernatant was generated from 293T cells transfected with pMDG (RRID: Addgene_187440) containing VSV-G envelope, psPAX2 (RRID: Addgene_12260) containing Gag, Pol, Rev, and Tat, and CAR containing construct pTRPE-3C10BBz by lipofectamine 2000 (Thermo Fisher Scientific, Cat#:11668030). Media was changed 24 hours after transfection, and the viral supernatant was harvested at 48- and 72-hours post-transfection. Viral particles were concentrated ~40-fold by ultracentrifugation for three hours at 4°C at 28,000 rpm with a Beckman SW28 rotor (Beckman Coulter).

### Generation of cDNA plasmid constructs encoding CAR T/ARMed CAR T

PD1 minibody sequence, leader-scFv-IgG1 hinge-CH3 were synthesized by GeneArt (Thermo Fisher Scientific) and then cloned into pTRPE lentiviral vectors or mRNA vector, pGEM.64A-based vector using XbaI & SalI restriction sites that were added to the PD1 minibody to facilitate moving to different vectors. Strep-tagII (WSHPQFEK) was added to the c-terminus of the Ab fragments by PCR.

### Isolation, electroporation, and expansion of primary human T lymphocytes

Isolated T cells were derived from leukapheresis products obtained from deidentified healthy donors under an institutional review board–approved protocol. T cells were stimulated with Dynabeads Human T-Activator CD3/CD28 (Thermo Fisher Scientific, Cat#:11141D) at a three-to-one, bead-to-cell ratio. T cells were cultured in RPMI 1640 medium supplemented with 10% fetal calf serum, Hepes buffer (20 mM), penicillin, and streptomycin (1%). The end of the first stimulation was determined based on a decrease in log-phase growth and a reduction of mean lymphocytic volume to 300 to 330 fL as measured on a Coulter Multisizer (Beckman Coulter), typically reached 10 days after stimulation, at which point cells are cryopreserved in FBS plus 10% DMSO.

### RNA CAR expression in primary human T lymphocytes

*In-vitro*-transcribed (IVT) RNA, the CAR-encoding gene constructs were subcloned into the pGEM.64A-based vector. mRNA was prepared using T7 mScript™ Standard mRNA Production System (Cell Script, Cat#:C-MSC11610). Using the BTX CM380 (Harvard Apparatus BTX) electroporation machine, IVT RNA was introduced into the T cells at a ratio of 1 µg RNA per one million T cells. Cells were rested at 37°C in 5% CO_2_ incubator for 24 hours before use for functional assays.

### Human T cell transduction and culture *in vitro*

Human T cell transduction and culture were performed as previously described (48). Briefly, isolated T cells were derived from leukapheresis products obtained from the Human Immunology Core at the University of Pennsylvania using de-identified healthy donors under an institutional review board-approved protocol. T cells were stimulated with Dynabeads Human T-Activator CD3/CD28 (Thermo Fisher Scientific, Cat#:11141D) at a 3:1 bead-to-cell ratio. After 24-hr stimulation, lentivirus was added into the culture media and thoroughly mixed to produce stably transduced CAR T cells. The concentration of the expanding human T cells was calculated on a Coulter Multisizer (Beckman Coulter) and maintained at 1.0–2.0 × 10^6^ per mL in RPMI-1640 plus 10% FBS serum (R10) media supplemented with 100 U/mL recombinant human IL-2 (rhIL-2; Proleukin, Chiron). Stably transduced human CAR T cells in the *in vivo* study were normalized to 30% CAR+ by addition of matched untransduced cells before transplantation.

### Mouse models

All mouse experiments were conducted according to Institutional Animal Care and Use Committee (IACUC)–approved protocols at the University of Pennsylvania.

For orthotopic models, 1 × 10^4^ D270-luc cells were implanted intracranially into 6- to 8-week-old female NOD-scid IL2Rgamma-null (NSG) mice (Jackson Labs), with 10 mice per group. The surgical implants were done using a stereotactic surgical frame and digital automated injector with tumor cells implanted 1 mm right and 1 mm anterior to the bregma, and 3 mm into the brain in 2 µL of PBS. Before surgery and for 3 days after surgery, mice were treated with an analgesic and monitored for adverse symptoms in accordance with the IACUC. For orthotopic experiments, animal survival plotted using Kaplan-Meier analysis, supplemented in some experiments by bioluminescent measurements as a surrogate for tumor volume, with tumor progression evaluated by luminescence emission on a Xenogen IVIS Spectrum following intraperitoneal D-luciferin injection according to the manufacturer’s directions (GoldBio, LUCK-2G).

Subcutaneous tumor models used 55.0 ×10^5^ U87-vIII-luc or D270 injected in 100 µL of PBS on NSG mouse flank. Subcutaneous tumor size was measured by calipers in two dimensions [length (L) & width (W)] for the duration of the experiment, where tumor size is reported as LxW (mm2) and tumor volume 0.5×L×W^2^ (mm3). Survival outcomes evaluated as Kaplan-Meier analyses.

For anti-PD1 Ab combination experiments, seven days after tumor injection, either subcutaneously or three days after orthotopically, mice were treated with a sub-optimal regimen of CAR T positive cells or negative controls in 100 µL of PBS delivered iv via the tail vein: 8 × 10^5^ on day 3 after orthotopic tumors, or 33.0 × 10^6^ on day 7 after subcutaneous tumors. One day after T cell injection and then every 4 days, 200 µg of anti-PD1 Ab (BioLegend, Cat#:329943, RRID: AB_2565904) was administered intraperitoneally.

For ARMed PD1 minibody experiments, mice were injected with 5 × 10^6^ CAR T ± minibody positive cells or negative controls in 100 µL of PBS iv three days after tumor injection for orthotopic, or 7 days after subcutaneous tumors. Survival was followed over time until predetermined IACUC-approved endpoint was reached which included limitations on tumor size or animal morbidity.

### Cell lines and culture

The human cell lines U87 and U87-EGFRvIII (U87-vIII) were provided by S. Chang (Northwestern University, Chicago, IL). Human GBM xenograft D270 was kindly provided by Dr. D Bigner at Duke University, Durham NC. These cell lines were lentivirally transduced to express the click beetle green luciferase and green fluorescent protein (GFP) under the control of the EF-1α promoter. At 48 hours after transduction, cells were sorted on an Influx cell sorter (BD Biosciences) based on GFP expression to obtain 100% GFP-positive cells that were then expanded and cryopreserved in FBS plus 10% DMSO. These cells were cultured in MEM (Thermo Fisher Scientific, Cat#:10373-017), no phenol red with 10% FBS, 20mM HEPES buffer (Thermo Fisher Scientific, Cat#:15630080), GlutaMax (Thermo Fisher Scientific, Cat#:35050061), 1mM sodium pyruvate (Thermo Fisher Scientific, Cat#:11360070) and 1% penicillin and streptomycin (Thermo Fisher Scientific, Cat#:15140122).

### Statistical analyses

All statistical analyses were performed using Prism (Graphpad) software. Differences between two groups were determined using an unpaired, two-tailed Student’s t test. For overall survival, groups were compared by Log-rank (Mantel-Cox) test. Error bars denoting S.E.M. Statistical significance was denoted by p-value as indicated, with non-significance (ns) determined as p>0.05.

## Data Availability

The raw data supporting the conclusions of this article will be made available by the authors, without undue reservation.
