# Supplementary material for: Checkpoint antibody receptor modified ARMed CAR T circumvents the suppressive immunome in GBM
Source: Front Immunol. 2025 Jul 31;16:1579925. doi: 10.3389/fimmu.2025.1579925 (PMC12351401; doi:10.3389/fimmu.2025.1579925)
Supplement: Supplementary file 1 [file SupplementaryFile1.docx]

**Supplementary Figure 1. Murine lymphocyte flow cytometry (A)** 13-parameter flow cytometry panel used to identify immune cell infiltrates in C57Bl/6 mice with GL261 experimental orthotopic implanted GBM. (**B)** Flow cytometric gating strategy for murine GBM-derived immune cell identification. **(C)** Bar graphs of differential immune cell infiltration in ipsilateral (RH) and contralateral (LH) brain hemispheres from weeks 1 through 4 from GL261-RH implanted GBM in C57Bl/6 mice. Statistical analyses were conducted using the multiple Mann-Whitney test, unpaired, non-parametric. Statistical significance was denoted as follows: P < 0.05 (*), P < 0.01 (**), P < 0.001 (***), and P < 0.0001 (****); P ≥ 0.05 was considered not significant (ns).

**Supplementary Figure 2. Human lymphocyte flow cytometry** **(A)** 13-parameter flow cytometry panel used to identify immune cell infiltrates in CD45-enriched and cryopreserved single-cell suspensions of resected patient GBM **(B)** Flow cytometric gating strategy for human GBM-derived immune cell identification.

**Supplementary Figure 3. GBM patient immune cell PD1/PDL1 staining (A)** Stacked bar graph comparing the average percent cellularity of PD1-positive cells within each immune infiltrate subset in human GBM tumor samples (n = 9), analyzed by flow cytometry. **(B)** Pie charts illustrating the average percentages of immune cells (live CD45-positive cells) in PBMCs and brain/tumor, normal donors (ND, n=7), and GBM patients (n = 8). **(C)** Percent of total cellularity of CD4+ versus CD8+ in brain samples from normal donors (ND, n = 6) and GBM patients (n = 9) and the percent CD45 of CD4+ versus CD8+ in PBMCs normal donors (ND, n = 7) and GBM patients (n = 8). **(D)** Stacked bar graph comparing the average percent cellularity of PD1-positive cells within each immune infiltrate subset in human GBM tumor samples (n = 9), analyzed by flow cytometry. **(E-F)** Stacked bar graph comparing the average percent CD45 of PDL1-positive cells within each immune infiltrate subset analyzed by flow cytometry (**E)** Normal donor PBMCs (n = 7) (**F)** GBM patient PBMCs (n = 8) **(G-H)** Stacked bar graph comparing the average percent CD45 of PD1-positive cells within each immune infiltrate subset analyzed by flow cytometry (**G)** Normal donor PBMCs (n = 7) (**H**) GBM patient PBMCs (n = 8). Statistical analyses were conducted using the multiple Mann-Whitney test, unpaired, non-parametric. Statistical significance was denoted as follows: P < 0.05 (*), P < 0.01 (**), P < 0.001 (***), and P < 0.0001 (****); P ≥ 0.05 was considered not significant (ns).

**Supplementary Figure 4. In vitro PD1-PDL1 CAR T functional inhibition assay** **(A)** PDL1 expression in human glioma cell lines with and without IFNɣ treatment (25 ng/mL for 24hrs). **(B)** Depiction of CD19 CAR T effector cells interacting with CD19+ PDL1+ K562 targets and expected signaling results. **(C)** The flow cytometry dot plot of CD19 versus. PDL1 staining on K562 WT (blue) and K562 CD19+PDL1+ (red), with negative staining control (secondary Ab only) in grey. **(D)** Multi-dimensional multi-color dot/histogram plots of T cells expressing the CD19 CAR or CD19 CAR and overexpression of PD1, where blue is CAR only, green is staining with anti-PD1, red is dual stain, and gray is secondary only. **(E-H)** Bar graph of intracellular cytokine detection by flow cytometry. The T cells shown in panel C were co-culture assays with target cells, shown in panel B. **(E)** Percent T cells that are IL-2+. **(F)** Percent T cells that are TNFα positive. **(G)** Percent T cells that are IFNɣ positive. **(H)** Bar graph of proliferation as measured by CFSE dilution and analyzed by flow cytometry for co-cultures of T cells, shown in panel D, and target cells, shown in panel C. Statistical analyses were conducted using the multiple Mann-Whitney test, unpaired, non-parametric. Statistical significance was denoted as follows: P < 0.05 (*), P < 0.01 (**), P < 0.001 (***), and P < 0.0001 (****); P ≥ 0.05 was considered not significant (ns).

**Supplementary Figure 5. In vivo murine model of PD1-PDL1 ARMed EGFRvIII CAR T inhibition in orthotopic D270 GBM xenograft in NSG mice** **(A)** Western blot of different fractions of StrepTacin bead purification of T cell supernatants and probed for StrepTagII run on SDS PAGE under reducing conditions, where the minibody monomer has a predicted size of 45 KDa. **(B)** Colorimetric ELISA PD1 binding assay using plates coated with recombinant PD1 or BSA negative control were incubated with 24-hour supernatants from cultured UTD or ARMed CAR T cells, secreting the anti-PD1 minibody. Conc. samples were concentrated before testing using StrepTacin beads. **(C-D)** Data from the D270 orthotopic GBM NSG xenograft mouse experiment in Fig. 5. **(C)** Graph of human T cell counts in peripheral blood lymphocytes of NSG mice in D270 IC ARMed CAR T model 18 days post-treatment. **(D)** Graph of human T cell counts in peripheral blood of NSG mouse in D270 IC ARMed CAR model 29 days post-treatment. **(E)** Diagram depicting PD1 ARMed CAR T secreting CPI mini-Abs and reversing checkpoint blockade and CAR T cell exhaustion in GBM. Created in BioRender. Cook, D. (2025) https://BioRender.com/pylxjs4.
